# Coming back together: a qualitative survey study of coping and support strategies used by people to cope with extended difficulties after the use of psychedelic drugs

**DOI:** 10.3389/fpsyg.2024.1369715

**Published:** 2024-05-28

**Authors:** Oliver C. Robinson, Jules Evans, David Luke, Rosalind McAlpine, Aneta Sahely, Amy Fisher, Stian Sundeman, Eirini Ketzitzidou Argyri, Ashleigh Murphy-Beiner, Katrina Michelle, Ed Prideaux

**Affiliations:** ^1^School of Human Sciences, University of Greenwich, London, United Kingdom; ^2^Challenging Psychedelic Experiences Project, London, United Kingdom; ^3^Division of Psychology and Language Sciences, University of College London, London, United Kingdom; ^4^Psychology Department, University of Exeter, Exeter, United Kingdom; ^5^Department of Psychology, Royal Holloway, London, United Kingdom; ^6^Department of Applied Psychology, New York University, New York, NY, United States; ^7^Perception Restoration Foundation, San Juan, Puerto Rico

**Keywords:** psychedelics, challenging experiences, adverse effects, qualitative, Structured Tabular Thematic Analysis, extended difficulties, integration

## Abstract

**Introduction:**

A growing body of literature is investigating the difficulties that some individuals encounter after psychedelic experiences. Existing research has explored the nature and predictors of these difficulties; however, a research gap exists in understanding how individuals endeavour to cope with such difficulties.

**Methods:**

The current study collected data from an international cohort of 608 participants who reported experiencing difficulties that persisted for at least one day after a psychedelic experience. They provided written data on how they used coping strategies to alleviate these difficulties. The qualitative analysis of the written data on coping was conducted using Structured Tabular Thematic Analysis.

**Results:**

A wide range of individual and social coping strategies were employed that were found helpful. The most common individual strategies were meditation and prayer, followed by self-educational activities such as reading and journaling. The most prevalent forms of social coping involved seeking support from friends or family members, followed by obtaining assistance from a therapist or coach. Features of social coping that were reported to be helpful included feeling heard/accepted, a non-judgemental attitude and sharing similar experiences.

**Discussion:**

Our findings hold potential for informing the design of therapeutic interventions and educational resources aimed at enhancing positive outcomes for those experiencing extended difficulties after psychedelic use.

## Introduction

Psychedelics are substances that have the effect of inducing a heightened or radically altered state of consciousness. They include LSD, psilocybin, mescaline, N,N-dimethyltryptamine (DMT) and ayahuasca, with other drugs like MDMA and ketamine also sometimes included in the psychedelic category. They exert their effects neurologically primarily through stimulation of the serotonin (5HT) 2A receptor (Nichols, 2016).

As psychedelics become increasingly explored as psychiatric medicines, there is a renewed interest in understanding their associated risks. Emerging data from clinical trials finds that up to 7% of participants report adverse psychological experiences lasting longer than a day with the use of psilocybin (Rucker et al., 2022) and MDMA (McNamee et al., 2023), including increased feelings of suicidality. However, researchers have suggested a potential underreporting of adverse events due to the absence of systematic assessments (Breeksema et al., 2022). Post-psychedelic challenges are higher in uncontrolled settings across the lifetime, as might be expected, with 8.9% of lifetime classic psychedelic users reporting functional difficulties lasting longer than a day (Simonsson et al., 2023) and 15% of those self-medicating with LSD or psilocybin mushrooms reporting negative psychological effects lasting beyond the acute effects of the drugs (Kopra et al., 2023). A survey by Bouso et al. (2022) found that 11.9% of ayahuasca users eventually sought professional mental health assistance for adverse effects. Another study, by Carbonaro et al. (2016), reported that among psychedelic users who encountered challenging experiences, 39% rated these as among the top five most difficult experiences of their lives. Furthermore, 7.6% of these individuals sought treatment for persistent psychological symptoms. Finally, in a recent survey of Norwegian psychedelic users by Kvam et al. (2023), 23% reported persisting adverse reactions lasting longer than a day, a fifth of which reported difficulties lasting longer than a year.

Evans et al. (2023) analysed brief narratives gathered from a large international cohort, all of whom reported extended difficulties following psychedelic experiences. The most commonly reported extended difficulties were general anxiety (as well as many sub-types of fear and anxiety such as ‘post-psychedelic trauma’ and ‘fear of going mad’); social disconnection; derealization and depersonalization; perceptual distortions; and existential confusion. Many of these narratives reveal existential and ontological struggles. Notably, about 30% of participants reported extended difficulties lasting over a year, with approximately 15% experiencing these challenges for more than 3 years. This study reported on the prevalence of types and subtypes of difficulty among a sample who all reported experiencing post-psychedelic difficulties lasting more than 1 day. Others such as Kvam, Simonsson, Bouso and Kopra et al. (2023), have reported prevalence of extended difficulties relative to psychedelic users overall. Kvam reported 23% of psychedelic users reported one or more adverse reaction lasting longer than a day, with sadness (4%), anxiety (3%) and headache (2%) being the three most common.

Despite this evidence of lasting unwanted psychological effects arising from challenging psychedelic experiences, little research has been conducted into how to best cope with or integrate such experiences, especially those with lasting negative effects. One study that has focused on this is Gashi et al. (2021), who interviewed psychedelic users who reported having a ‘bad trip’ characterised by feelings of losing oneself, going crazy, or ego dissolution. They concluded that participants would construct their own personal *bad trip stories*; narrative accounts of the episode that allowed them to cognitively make sense of the experience and find good outcomes among the difficulties, and thus turning them into meaningful events, while also integrating aspects of their life history or current relationships that required some kind of narrative resolution too. Such narratives allow individuals to comprehend and assimilate their distressing encounters into their personal life stories (González et al., 2022).

Integration has been defined as a process following psychedelic experiences in which important lessons from the experience are discerned and integrated into the daily life of the person in a way that allows them to live a fuller, less stressful life (Richards, 2017). It is commonly included as one of the three stages of psychedelic-assisted therapy – preparation, the drug experience, and integration – with most clinical trial treatment protocols including one or two sessions of integration after the drug session (Thal et al., 2023). These treatment protocols typically do not anticipate and are not designed to treat problems that may emerge during or after a psychedelic experience (Jacobs et al., 2023). Integration can involve short-term challenges (Lutkajtis and Evans, 2023), and can be supported by connecting with a community that helps make sense of an experience (Earleywine et al., 2022). It may also involve the ongoing integration of profound shifts in world view (Timmermann et al., 2022), metaphysical challenges (Sjöstedt-Hughes, 2023) or ontological ‘shock’ (Breeksema and van Elk, 2021).

Such metaphysical and ontological challenges leading to profound shifts in worldview can often result in spiritual reorientations. Sometimes this process can be turbulent and disturbing – such instances have been called ‘spiritual emergencies’ (Lukoff, 1985; Grof and Grof, 1989). Features of ‘spiritual emergencies’ may include feelings of being overwhelmed, confused and challenged by perceptual and worldview shifts (Grof and Grof, 2017), as well as transpersonal experiences such as psychic-like experiences (e.g., telepathy), out-of-body experiences (Luke, 2022) and visual and auditory hallucinations of a religious or archetypal/symbolic nature (Hansen and Brodtkorb, 2003). Practices reported to help cope with non-psychedelic-induced transformative experiences include breathwork techniques, mindfulness practice, consulting spiritual literature, yoga, lucid dreaming, nature immersion, and athletic activity. The majority (84%) of those surveyed reported benefit in managing their experience, whereas 7% reported no benefit, and 3% reported a worsening of symptoms (Evans and Read, 2020; Brook, 2021; Corneille and Luke, 2021). It’s worth noting that ‘spiritual emergency’ is not a common term in mainstream psychology or psychiatry and is confined to the relatively niche context of transpersonal psychology.

There is a wide range of possible therapeutic approaches to psychedelic integration (Thal et al., 2023), many of which indicate the importance of psychological flexibility (Hayes et al., 2011), including but not limited to Acceptance and Commitment Therapy (ACT) and the Acceptance, Commitment and Embody (ACE) model (Watts and Luoma, 2020) for those using psychedelics for mental health treatment. Focusing on the metaphysical and existential challenges often arising from psychedelic experiences, some have proposed more existential (e.g., Carnahan, 2023) or transpersonal (e.g., Papaspyrou, 2015) therapeutic approaches to integration, whereas others also note the potential benefit of somatic practices, such as yoga and breathing techniques (Gorman et al., 2021). However, to date, there has been no controlled comparative research into different models (Thal et al., 2023). Furthermore, while there is much in the literature on how to deal with challenging experiences in the acute phase of a psychedelic experience, there is very little research on what helps people cope with lasting post-psychedelic difficulties.

The coping theory that frames this study stems from the transactional theory of stress and coping, developed by Lazarus and Folkman (1987). This theory posits that stress is the outcome of a cognitive appraisal of a real or imagined transaction between a person and their environment perceived as threatening or harmful, and that coping is aimed to rectify this and the negative emotions. There are two levels of cognitive appraisal: primary appraisal involves an evaluation as to whether a current or future situation has the potential for harm, while secondary appraisal involves an evaluation of whether the individual possesses the coping resources to successfully handle the situation. Coping is an umbrella term that refers to ways of dealing with stressful person-environment transactions (Stanislawski, 2019). Coping strategies can be distinguished by way of a set of binary distinctions, including; (a) problem-focused or emotion-regulation focused, (b) active or avoidant, and (c) proactive or reactive. Establishing whether coping strategies are effective or not requires looking at them in the context of the stressor and establishing if they are helping the individual solve the problem in question or regulate their emotions, and if so, over what timeframe. Coping is by no means always an individual process. Social support is a widely researched form of coping that provides two recognised potential ways of alleviating stress; *instrumental* social support provides support with solving a problem, while *emotional* social support helps with the emotions associated with the stress (Kent de Grey et al., 2018).

### Aims and research questions

Despite two decades of renewed interest in psychedelic research, there remains a significant gap in understanding how individuals respond to and cope with extended post-psychedelic difficulties, and which coping strategies are perceived as adaptive and beneficial. Addressing this research void, the current study aims to explore the personal and social strategies that individuals find helpful in managing extended difficulties after psychedelic drug use, through the collection of brief personal narratives elicited from individuals who all report difficulties lasting a day or more after psychedelic experiences. A key contextual issue that inevitably frames such narratives is that many individuals who take psychedelics will do so in cultures where it is illegal to do so, meaning the requirement for a certain amount of concealment, secrecy and the potential dangers of being found guilty of a crime or being shamed by others who do not approve. The attempts at coping described and reported below are, for the majority of the sample, conducted in such environments. With that context in mind, the study is guided by two primary research questions:

1. What are the types and narrative descriptions of coping strategies used by individuals who report having a psychedelic experience that they believe led to difficulties lasting more than one day?
2. What sources, forms and features of social support were experienced as helpful in coping with extended difficulties following a psychedelic experience?

## Method

### Design

This study employed a cross-sectional, single-phase, convergent mixed-methods survey design. The approach to qualitative data collection and analysis adopted in the study – use of a large sample with brief written narrative data – sits within the critical realist epistemological ethos of Structured Tabular Thematic Analysis (described in more detail below) (Robinson, 2022). See Evans et al. (2023) for a thematic analysis of data from the same survey on the difficulties encountered after the psychedelic experience. The data presented in the current article relate specifically to the personal and social coping strategies used that were found to be helpful in alleviating difficulties. The nature of the retrospective, narrative data collected means that the causal link between the psychedelic experience and difficulties is inferred by the participant, and no claims as to the causal link between the former and the latter can be made.

As a preliminary qualitative investigation into this under-studied area, this report analyses the sample of participants as a single cohort, bound together by the commonality of having experienced personal difficulties after a psychedelic experience, without breaking them into demographic groups or groups defined by sociocultural context or by the circumstances, set or setting of the experience. Such group differences are better suited to quantitative analysis (*cf.* Evans et al., 2023, for example, in comparing difficulties across guided/unguided context of the psychedelic experience using quantitative methods). This form of group-based quantitative analysis will be subject of continued further studies.

### Participants and recruitment

For inclusion in this study, participants were required to meet three criteria: they must have experienced difficulties immediately following the use of a psychedelic drug, with these difficulties significantly impairing their functioning for more than a day after the drug’s pharmacological effects subsided. Additionally, participants had to be aged 18 or older and possess proficient or fluent English language skills. The recruitment strategy was multifaceted, involving the dissemination of the survey link through various channels including social media, an online newsletter, email lists targeting students, newspaper advertisements, and a research participation list. No financial incentives were offered for participation. A total of 608 individuals completed the survey between October 2022 and January 2023. Demographic frequencies of the sample are detailed in Table 1.

**Table 1:** Frequencies and percentages of demographic categories within the dataset.

		Frequency	Percentage of sample
Gender	Female	298	49%
	Male	292	48%
	Other Gender	12	2%
	Not stated	6	1%
Age	Aged 18–24	67	11%
	Aged 25–34	176	29%
	Aged 35–44	158	26%
	Aged 45–54	116	19%
	Aged 55+	85	14%
	Age not stated	6	1%
Educational level	High school educated	91	15%
	Undergraduate degree	200	33%
	Masters degree	195	32%
	PhD or other doctoral degree	61	10%
	Other / prefer not to say	61	10%
Nationality	USA	197	32%
	British	136	23%
	Canadian	25	4%
	Other (45 countries represented in total, with each max 2% of sample) – see footnote	250	41%
Ethnicity	White	505	83%
	Hispanic	18	3%
	Mixed ethnicity	31	5%
	Asian	6	1%
	Other	24	4%
	Not stated	24	4%

Countries in sample other than UK, USA, and Canada: Austrian; Australian; Belarusian; Bulgarian; Belgian; Brazilian; Colombian; Croatian; Czech; Danish; Dutch; Egyptian; Filipino; Finnish; French; German; Greek; Hungarian; Indian; Iraqi; Irish; Israeli; Italian; Lebanese; Lithuanian; Mexican; Maltese; New Zealander; Norwegian; Pakistani; Portuguese; Polish; Romanian; Russian; Serbian; Slovenian; Spanish; Swiss; South African; Swedish; Turkish; Tibetan; Ukrainian; Uruguayan; Venezuelan.

### Researcher characteristics

The design of this study was devised by an international, interdisciplinary team across multiple institutions, with specialisms in psychology, psychiatry, philosophy and the humanities. Analysis of the data was conducted by three researchers: an academic specialising in the integration of philosophy and psychology (Analyst 1), a psychologist with specialism in qualitative methods and mixed methods (Analyst 2), and a psychology researcher with an expertise in psychedelic science (Analyst 3).

### Data collection

The study gained ethical approval from the University of Greenwich Research Ethics Board prior to the commencement of data collection (application ref: 21.5.7.20). The online survey platform Qualtrics was used to collect data. The questionnaire comprised a series of open-ended and closed-ended questions. The instructions provided to participants to describe in written form the coping strategies and sources of support they found helpful were as follows: (1) “If you used coping strategies that you found helpful in dealing with the difficulties/challenges after the trip, please describe these in a short paragraph.” (2) “Did you seek support from other people to help with the difficulties you experienced? If you found some or all of the support from others to be helpful, please describe what you found to be most helpful and why.”

Participants were shown a debrief form at the end of the questionnaire, which provided information about support organisations and information websites that are orientated towards supporting individuals who have experienced difficulties with psychedelics and psychedelic integration.

### Qualitative analysis

Structured Tabular Thematic Analysis (ST-TA) was employed to analyse the data (Robinson, 2022). This recently developed form of thematic analysis is specifically designed to analyse brief texts, for example comments in social media feeds or answers elicited by open-ended questions in questionnaires. It uses spreadsheet software to organise the data and thematising. ST-TA is influenced by the reflexive thematic analysis of Braun and Clarke (2006), and also the ecumenical approach to thematic analysis devised by Boyatzis (1998). Alongside rich description of themes and patterns, it includes an additional focus on theme frequencies. Brief text data permits larger sample sizes than more in-depth qualitative approaches, within an equivalent resource envelope, and frequencies are more meaningful with higher participant numbers (Robinson, 2022).

Given the lack of extant qualitative or mixed method research on the topic of coping with extended post-psychedelic difficulties, we chose to conduct an inductive analysis for the current study. The phases for inductive analysis in ST-TA are as follows: (1) Deep Immersion in the Data; (2) Generating Initial Codes and Themes; (3) Tabulating Themes Against Data Segments; (4) Checking Inter-analyst Agreement; (5) Exploring Theme Frequencies, (6) Developing Thematic Maps and Diagrams; and (7) Producing the Report (Robinson, 2022).

With regards to checking and developing agreement between analysts, this is part of ensuring that themes are transparently and cogently described and labelled, and of supporting eventual conclusions as consensual and not based on idiosyncratic interpretations pertaining to an individual researcher. ST-TA employs the agreement-reaching process to ensure that themes are sufficiently cogent and transparent for multiple analysts to code independently. The aim is to reach 80% agreement (Robinson, 2022). Using randomly selected samples of responses and analyst pairs for the analysis of individual coping data, it took three iterations of agreement check to reach 80%. For the analysis of social coping data, it took two iterations to reach 80%.

## Results

**Figure 1:**
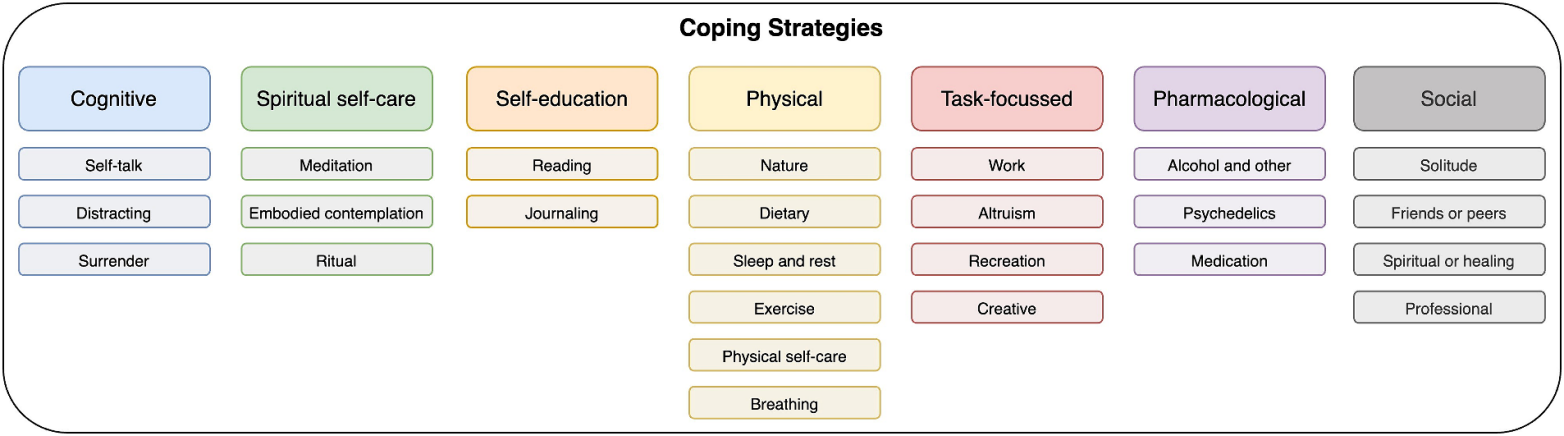
A diagrammatic presentation of all coping meta-themes and sub-themes (abbreviated labels used – see Tables 1, 2 for full theme labels).

This section presents the thematic analysis of coping strategies and sources of support that participants described as being helpful in managing extended difficulties following psychedelic experiences in short written narratives. There are eight meta-themes and within each of these there are subthemes. The frequencies with which these themes were represented in the data are shown in Figure 1.

Tables 2, 3 show the full list of meta-themes and sub-themes with frequencies and percentages of the sample. A brief summary of most prevalent themes: The most common individual coping strategy was ***Meditation and prayer***, followed by ***Reading*** (self-educational) and ***Physical exercise***. The most common type of social coping was connecting with ***Peers and community support***, including family and friends, followed by **Professional therapeutic or coaching assistance**. The most prevalent feature of social coping found to be helpful was the acting of ***Talking and feeling heard*** by others, followed by ***Acceptance and validation*** and ***Shared similar experiences***. These and all other themes are described in full with example quotes below.

**Table 2:** Meta-themes and sub-themes for all themes except social coping themes, with number of participants (and corresponding percentage of sample) for whom subthemes apply (*N* = 608).

Meta-theme	Sub-theme	Number of participants	Percentage of sample
Cognitive coping	Self-talk / affirmations	52	9
	Meta-cognitive distancing	22	4
	Acceptance and surrender	62	10
	Distraction / external focus	23	4
Spiritual self-care coping	Meditation and prayer	161	27
	Embodied contemplative practices	63	10
	Ritual practices	4	1
Physical coping	Physical Exercise	74	12
	Physical self-care techniques	32	5
	Time in nature	62	10
	Body relaxation	35	6
	Breathing strategies	64	11
	Sleep and rest	27	4
	Diet-based strategies	51	8
Self-education strategies	Reading	88	14
	Journaling	69	11
Task-based coping	Recreational strategies	37	6
	Creative strategies	28	5
	Work-based coping	47	8
	Altruistic tasks	11	2
Pharmacological coping	Further use of psychedelics	42	7
	Use of alcohol or other recreational drug	37	6
	Psychiatric medication	42	7
	Abstaining from particular substances	32	5
No coping strategies found helpful	N.A.	6	1

**Table 3:** Social coping meta-themes and sub-themes, with number of participants (and corresponding percentage of sample) to whom subthemes apply (*N* = 608).

Meta-theme	Sub-theme	Number of participants	Percentage of sample
Types of social coping	Professional therapeutic or coaching assistance	107	18
	Spiritual and traditional healing guidance	39	6
	Peers and community support	209	34
	Solitude and Isolation	39	6
Emotional support – helpful qualities	Non-judgemental attitude	35	6
	Patience and lack of pressure	10	2
	Acceptance and validation	88	14
	Feeling loved	12	2
	Talking and feeling heard	127	21
	Shared similar experiences	81	13
	Honesty	8	1
Instrumental support – helpful qualities	Facilitating self-understanding	60	10
	Helping make sense of experiences	85	14
	Financial support	2	1
	Support with day-to-day tasks	11	2
	Providing access to medicines	11	2

### Cognitive coping strategies

This theme subsumes all cognitive coping techniques for managing thoughts and interpretations during the extended difficulties. The coping techniques employed are similar to those employed in Cognitive Behavioural Therapy and Acceptance and Commitment Therapy, but were used by participants without the intervention of a therapist. There are four subthemes. The first subtheme is ***Self-talk and affirmations**,* which relates to positive or compassionate self-talk / inner dialogue, positive affirmations. One participant wrote “I was also dialoguing with myself and reassuring myself,” while another wrote “Reminding myself I was a caring responsible loving person.” The second subtheme is ***Distraction and external focus***, which relates to placing attention intentionally on other things. For example: *“*Just trying to not notice/focus on it. That’s the best way I’ve found to get through it.” The third subtheme is ***Acceptance and surrender**.* This includes all reports of feeling helped by cultivating an attitude of surrender and acceptance, for example “My number one coping strategy was trust. Trusting that my life journey is happening just as it’s meant to, and that everything will work out. Trust that I am supported by the universe.” The fourth subtheme is ***Meta-cognitive distancing*** for example “Observing thoughts and emotions,” and “Training myself to detach my emotions from my destructive thoughts.”

### Spiritual self-care strategies

This theme includes coping methods explicitly drawn from spiritual traditions, such as various forms of meditation, prayer, yoga, tai chi and ritual practices. There are three subthemes. The first subtheme, by some distance the most frequently stated by our participants, is ***Meditation and prayer**.* Sometimes respondents described specific types of meditation, such as loving-kindness meditation, or mindfulness, and other times they simply wrote ‘meditation’ without giving details. For example: “Mindful meditation in which I focus on an imaginary fixed point. And when a thought pops in my head, I acknowledge it, without resisting it and then gently shift my focus back to that point.” We also included prayer and chanting in this sub-theme, which people used in a variety of ways, from inducing somatic changes to cultivating attitudes of trust and surrender: “The coping strategy I use the most is prayer. Praying for the outcome that God’s will prevails and I do not get too hung up on my own perception of results.”

The second subtheme is ***Embodied contemplative practices**.* Participants reported the effectiveness of spiritually-oriented movement practices like yoga, tai chi and ecstatic dancing practices in the process of coping with their difficulties, especially as a way of changing their mental state through bodily movement. “I had already been a yoga practitioner for many years and sticking with my yoga practice helped immensely – getting out of my head and into my body has definitely been one way to escape the paranoid thoughts.” The third subtheme is ***Ritual practices**.* This subtheme represents all the religious and spiritual rituals mentioned as a way to find calm and navigate the difficult experiences. For example “Grounded myself with focus on my senses, chanted, and prayed to gods and did rituals that soon unmasked themselves as an effective gestalt technique to resolve my issues. Writing, transformation of intent into symbols and doing meaningful rituals to achieve mental and emotional stability, success in my endeavours, resolution of problems etc.”

### Physical coping practices

This theme includes practices focused on the physical body, other than those within the embodied contemplative practices sub-theme. These practices were described as helping participants ground themselves in their body and regulate their nervous system. There are seven subthemes.

The first subtheme is ***Physical exercise***. Participants highlighted the helpfulness of physical exercise, ranging from running to cardiovascular workouts to strength training, for alleviating psychological distress, promoting mental clarity, and reestablishing connection with their physical bodies. For example: “I’ve tried therapy, meditation etc., but it has not done much. The only time I feel somewhat good is when I lift weights.” The second subtheme is ***Physical self-care techniques**.* This includes responses that mentioned acupuncture, massage, showers and bathing. Several respondents reported finding bathing helpful in the aftermath of a challenging psychedelic experience, either for relaxation or for reconnecting to the body. For example: “In the immediate aftermath, cold showers, no caffeine and to-do lists (chores, etc.) were tremendously helpful…cold showers helped me feel grounded, and maybe “calibrated” my nervous system to cope with higher levels of stress.”

The third subtheme is ***Body relaxation**.* Participants mentioned non-specific relaxation exercises, such as self-soothing, and stress-management by taking time out: “Tried to be easy on myself, which is not something I am the best at. Allowing my body to relax and feel all the anguish and torment and it was like I slowly digested all of it.” The fourth subtheme is ***Breathing strategies***. This subtheme includes the variety of breathing techniques employed by participants including deep breathing, Wim Hof method ®, box breathing, circle of life breathing, Holotropic Breathwork ®, or non-specific breathing methods. These were particularly mentioned in the context of relieving anxiety and stress. For example: “Breathing: Using a repetitive mantra: R – breathing the letter out as a hum, E – breathing the letter out as a hum, L – breathing the letter out as a hum, A – breathing the letter out as a hum, X – breathing the letter out as a hum, then saying the whole word ‘RELAX’ – repeat as long as necessary until relaxed.” The fifth physical coping subtheme is ***Sleep and rest***. Some respondents reported turning to sleep or rest as recovery methods in the immediate aftermath of a challenging psychedelic experience: “I rested a lot. I was exhausted like I have never been in my life.”

The sixth subtheme is ***Diet-based strategies***. Many participants found dietary changes, supplements and cleanses beneficial, helping them shift to a healing mindset and strengthening their bodies: “It was important to do all the healthy things as well. Exercise, healthy eating, following my life’s path and connecting to a future that excites me when I can.”

The final subtheme is ***Time in nature***. This was reported to have therapeutic benefits, underscoring the restorative potential of the natural environment. For example, one participant wrote “Tried to minimise stress in my life…Tried to spend time in nature, deleted news apps and social media from my phone and spend time doing things that made me feel more grounded.”

### Self-education strategies

This theme relates to attempts at learning and self-educating, via reading and journaling, to help solve the difficulties being experienced. Participants found that active learning, critical reflection and information-seeking were helpful coping mechanisms to help them make sense of their psychedelic experience and the after-effects. There are two subthemes.

The first subtheme, ***Reading***, was focused on non-fiction that could help with learning about the problems in question, including psychoeducation about anxiety, panic, trauma responses, and the autonomic nervous system. For example: “I did not realise that I was having a panic attack until several days later, I just knew it was a terrifying emotional and perceptual experience. I looked it up on the Internet and realise that it could likely be associated with the MDMA. Just knowing that made me feel a little less terrified, and I never had a panic attack as severe again.”

A prevalent feature in this subtheme was the therapeutic value of finding similar stories of post-psychedelic crisis and recovery: “I found labelling the feelings as ‘anxiety’ very helpful, and reading about people who had had similar experiences and found ways to manage them.”

People reported finding research on adverse meditation experiences helpful. And they turned to sites like Reddit and Erowid for information on challenging psychedelic experiences and enduring difficulties like HPPD. Longer-term, people also turned to spiritual and philosophical literature to try and make sense of their experience and lessen the existential or spiritual confusion they felt. For example: “People like me need some kind of integrative philosophical framework to make sense of the psychedelic experience, in both its negative and positive aspects. Listening to Alan Watts was a big help.”

Some people mentioned specific books they found helpful to their recovery. The most commonly-cited author was transpersonal psychologist Stanislav Grof. For example, one participant wrote: “While driving to and from work, I began listening to Stan Grof’s audiobook: The Way of the Psychonaut. Many things he said made sense to me and finding phrases like “spiritual emergency” to describe my experience helped me see that I wasn’t completely losing my mind.”

The second subtheme, ***Journaling***, relates to descriptions of finding benefit in journaling or writing for the purpose of self-directed learning and reflection, promoting processing and integration of psychedelic experiences. For example: “I was journaling a lot, even if nothing made sense to me I kept writing and writing, day after day until I could finally see some patterns and understand.”

### Task-based coping strategies

These approaches underscore the therapeutic value of engaging with task-based activities which refocus the mind away from disturbing internal thoughts or emotions towards external activities, such as work, creativity, gaming, altruistic activities or time in nature. These expand on the cognitive coping subtheme of Distracting and refocusing. There are five subthemes.

The first subtheme is ***Creative strategies***. Artistic expression was identified as a therapeutic tool in post-psychedelic recovery by several participants, especially making art, creative writing and playing music. For example: “I tried to maintain a daily creative writing practice to keep my mind from focusing on what was coming up from the trip.” The second theme, ***Recreational strategies**,* included responses that mentioned gaming or listening to music as distraction and relaxation techniques. For example: “I played classical music nearly constantly for the next 24 h, even during the few hours of sleep that I had.” Thirdly, ***Work-based coping*** was mentioned as beneficial by some respondents, for example focusing on their job, or doing domestic work and chores. This focus on work helped to convey a sense of normality, routine and structure. An example quote in this theme is “I was able to cope further by throwing myself into work: self-studying into a new career as a software engineer. This may have saved my life.”

Fourthly, ***Altruistic tasks**:* Some respondents found that actively helping others was the best means of coping with their own difficulties, and initiating projects with such an aim. For example: “I started a network for integration for people who receive ketamine therapy at home. I am trying to establish a culture around psychedelic therapy that resists being hijacked by toxic positivity and individualism.”

### Pharmacological coping

This theme includes all references to the helpful continued use of psychedelic or non-psychedelic drugs, or conversely abstinence in relation to specific substances. The first subtheme is ***Further use of psychedelics**.* Some respondents said that trying psychedelics again after their challenging experience helped them to integrate the initial adverse experience. For example: “Most helpful was tripping again to “work it out” where I went wrong. The state just persisted until I took ayahuasca.” The second subtheme is ***Use of alcohol and recreational drugs**.* Some survey respondents said that alcohol, cannabis and other recreational drugs helped them to cope with post-psychedelic anxiety and trauma, although some felt this was a short-term coping technique which did not help in the long-term: “What was helpful at the time with coping was excessive use of alcohol and other drugs. An immediate but not long-term coping strategy however as years later I entered a treatment program in rehab for substance abuse. So not a healthy coping strategy really.”

The third subtheme is ***Psychiatric medication**.* Some participants reported being helped by psychiatric medication, such as antidepressants and antipsychotics. For example, one participant wrote “what turned out to help was plain old antidepressant meds. It took 10 months to get the right cocktail but when it worked, it worked. It was pretty evident it was the meds and not anything else.” The fourth subtheme is ***Abstinence from particular substances**:* This theme relates to mentions of giving up substances such as psychedelics, cannabis, alcohol or caffeine as methods for regulating the nervous system and returning to healthy functioning, for example “No alcohol, drugs or caffeine at all” and “I swore off psychedelics for a long time.”

### Types of social coping

Participants identified particular sources of social support who were helpful or unhelpful. These were diverse and included professional assistance, spiritual guidance, and peer and community support. It is notable from the responses that people experiencing post-psychedelic difficulties are likely to encounter very different diagnoses of their problems if they go to a counsellor, a transpersonal psychotherapist, a psychiatrist or a shaman or guide. Some sources of support may interpret post-psychedelic difficulties as ‘spiritual emergencies’ which should be allowed to happen but given support, others as neurochemical problems requiring medication.

The first subtheme is ***Professional therapeutic and coaching assistance***. Participants indicated that they sought assistance from trained professionals who provided an expert, structured approach to coping. This support, offered by therapists, psychiatrists, coaches, and other professionals like nurses or professors, involved scientifically backed strategies like Cognitive Behavioural Therapy (CBT), Somatic Experiencing, Internal Family Systems Therapy, and Jungian psychotherapy. Participants found these structured approaches beneficial, providing them with concrete tools and frameworks for processing their experiences and managing their reactions. One example quote is: “I had CBT therapy on the NHS about 2 years after when I was having flashbacks once a week. Surprisingly, it helped loads recognising that essentially I was catastrophizing.” Another example is: “The most important was therapy. My therapist helped me rearrange all the things that came up during the trip and understanding them differently.”

Finding a therapist that is familiar with psychedelics and post-psychedelic difficulties was frequently described as important: “I first had a chat with a counsellor which did not help all that much (he had little experience with psychedelics).” However, some respondents said that more psychedelic-positive therapists or counsellors could sometimes over-spiritualize their difficulties, for example: “I reached out to counsellors who help integrating psychedelic experiences, with mixed results (after a session with one person I actually felt worse, as the notion of karma was proposed as an explanation of my experience).”

The second subtheme is ***Spiritual and traditional healing guidance***. Participants reported drawing on the guidance and support of spiritual leaders and traditional healing practices, underlining the significance of spirituality and tradition in their mental health recovery journeys. For example: “Working with a shaman and plant medicine community provided a group of peers who had also worked through repressed memories, specifically that of sexual abuse by family members. That validation is hard to understate as the skepticism from many professionals and my inner circle was devastating. Working with a skilled shaman and a dieta [shamanic diet] was second only to my wife in providing relief, understanding, and ultimately healing.”

However, again, some respondents complained that spiritual guides sometimes downplayed or over-spiritualized their difficulties. For example: “My guide was super helpful, but also such a believer in the power of the medicine it’s hard for him to frame anything as ‘bad’.”

The third social support subtheme is ***Peers and community support***. This was the most common type of coping technique mentioned. This type of support came from friends, family, online communities, or others who have shared similar experiences. Respondents emphasised the importance of empathy and shared experience in the support process. For example: “My partner told me that everything will turn out okay, and sometimes it’s really hard to believe it in the moment when you are going through terrible feelings, but it chilled me out a little, he hugged me and kept me close. A friend of his (and mine) convinced me to seek therapy and that gave me hope to be okay, and it did.”

Again, respondents said that the responses of friends and family were not always helpful, underlying the challenge posed by a lack of wider public understanding of post-psychedelic difficulties. For example: “support from family and regular friends (non-psychedelic) was almost useless. I felt like I was existing in a totally different terrain than “normal” people.” Also, in this subtheme, psychedelic integration circles were mentioned as a peer-group environment that some found helpful. For example: “I joined a group for psychedelic integration and that helped HUGELY! I consider myself pretty self-aware and being able to ‘talk it out’, share my process and being met with understanding and non-judgement made a difference. The other thing that helped was realising that the experience itself does not “fix” anything. Integration is EVERYTHING!”

Finally, we included ***Solitude and isolation*** as a social coping subtheme. While many participants found it helpful to reach out to others, some also mentioned they found it helpful to reduce social contact, including taking time off work, to give their nervous system a chance to calm down: “I think the best option I had was to stay home in a quiet isolated place with basic food and rest.”

### Emotional support – effective qualities

The Emotional support meta-theme includes the qualities shown by supportive others that were perceived to be helpful, other than instrumental guidance. Eight subthemes sit within this meta-theme. The most common subtheme in this theme is ***Talking and feeling heard***. Participants described that being able to verbalise their experiences and simply feeling heard was therapeutic. This process offered them a form of emotional release and validated their experiences. For example: “The most helpful has been group work. The opportunity to be seen, heard, and accepted by a group of others who are interested in my well-being has helped increase my sense of connectedness and safety.”

The second most common subtheme is ***Acceptance and validation**.* Being accepted, validated, and reassured by others fostered a sense of self-worth and helped them navigate through their mental health challenges with greater confidence. These elements affirmed and acknowledged their experiences. For example: “Having other people that have been in similar trips to give me reassurance that I wasn’t going crazy was important.”

The third subtheme is ***Shared similar experiences*** with others. It was described how this fostered a sense of camaraderie and understanding. Knowing that others faced similar challenges provided comfort and offered unique insights or advice. Examples of this subtheme included the following: “Most helpful were people who have worked through similar experiences and helped me see where my Mind went wrong.” And also: “I think it’s so important to have friends with similar experiences. I think there’s an intense desire to talk about these experiences in order to make sense of them.”

The fourth subtheme is ***Non-judgemental attitude**.* Participants described a non-judgemental attitude as enabling a sense of acceptance and understanding. The absence of criticism or ridicule in such interactions allowed them to openly communicate their experiences. For example: “Non-judgement about me, the experience or my reaction. Validating and expressing compassion about what it was like for me. Respecting the meaning I gave the experience, and not insisting upon their interpretation.” The fifth subtheme is ***Feeling loved**.* More than merely being accepted, some participants said that it was feeling loved by the people around them that helped them stabilise and recover after a challenging experience. For example: “My wife sacrificed a year of her life to care for me. It’s impossible to describe love like that in a survey.”

The final two sub-themes also relate to a sense of openness and trust. ***Patience and lack of pressure***; participants described how patience and a lack of pressure from their support person created a safe space in which they could express their experiences and emotions freely, processing their experiences at their own pace. For example: “Patience with me being forgetful was very helpful.”

The final subtheme of emotional support is ***Honesty***. Some participants identified honesty as a vital element in building trust within the support process. The transparent communication from their support system fostered an environment where they felt safe to express themselves authentically. For example: “Had a chat with a friend of mine who also takes psychedelics as healing and spiritual tools – someone who I can be completely open with.”

### Instrumental support – effective qualities

Instrumental social support as a meta-theme subsumes all mentions of receiving support with day-to-day tasks or more existential tasks such as facilitating self-awareness and insight into the experience. This overlaps with the provision of emotional social support but is more solution-focused and functional in its description. In terms of day-to-day tasks, one subtheme is ***Financial support***, which was described as especially helpful when post-psychedelic difficulties made it hard for them to work. There was also **Support with day-to-day tasks** when their post-psychedelic difficulties made it hard to function normally, for example offering reminders to perform tasks in the context of memory issues: “I was having issues with memory at the time, so my friends at Sixth form made sure that I ate my lunch, and attended my lessons. I would frequently have naps during the day so they knew my timetable to make sure I got to my lessons on time and would walk with me to the lesson to make sure I did not get lost or distracted.”

In addition, as an element of instrumental social support, individuals helped with ***Providing access to medicines*** (both plant and conventional medicines) that the individual was unable to get for themselves – this was mentioned as contributing to effective coping.

In terms of instrumental help focused on helping with insight or understanding, participants noted that some social interactions helped with ***Facilitating self-understanding***. In these social environments they were actively encouraged to explore and understand their thoughts, emotions, and behaviours, contributing to their personal growth. For example: “I also talked to a friend on phone who helped me by just being my very old friend making me remember myself.” Finally, participants found that that certain kinds of social support were integral in ***Helping make sense of experiences***. The assistance they received in making sense of their experiences helped them integrate these experiences into their broader life narrative. Here is one such narrative of this sense of being helped to make sense of experiences by others:

“The greatest support came by way of conversations with my therapist and friends. It helped to see a friend who tripped with me report similarities in his agnosticism and sense of uncertainty in his worldview. In addition, I had a conversation with a coworker almost 7 months later who helped me realise that the place my mind went to was a real psychosocial space describing a real part of my mind. At the time I just wrote off the experience as a terrible trip because the dose was much higher than I was prepared for, and the setting was too exposed to strangers wandering by at a campsite. While that may have been a very true possibility, I think I learned that I may have a hyperactive superego that was wrapped up in my religious upbringing. So discussing the experience with other people I trusted has been critical, and I’m still making sense of what the hell exactly happened.”

### No coping strategies found to be helpful

Finally, this meta-theme gathers the spontaneous comments provided in which participants say that they found nothing to be helpful in dealing with the enduring difficulties. Example descriptions of this lack of coping strategies are as follows: “I’ve been in the mental health system since February of ‘99. Nothing has helped & in many ways got worse. I feel so defective & broken. Every day I question my life & my future. Whilst unable to escape the horrors of my past. It is truly hell on earth.” Another example quote is: “At the time, many years ago, I had few coping abilities to deal with this. I just groped around at random to try to find something, without success.”

## Discussion

This research study aimed to explore what helped individuals cope with extended difficulties after psychedelic experiences. Our findings indicate that both social support and self-management techniques are components of helpful post-psychedelic care, when navigating extended difficulties. The most frequently used helpful coping technique was seeking support from a friend, family member or peer group. This accords with Cowley-Court’s finding that ‘a frequently cited tool to support integration was sharing with like-minded others or communities’ (Cowley-Court et al., 2023). Another popular coping technique was talking therapy and counselling, which aligns with Aixalà’s (2022) recommendation for the integration of difficult trips. Insights were also gleaned concerning what individuals found beneficial in the realm of social or professional support. Respondents underscored the significance of attributes such as non-judgementalism, acceptance, patience, and a compassionate approach in their support networks. Additionally, it was highlighted that avoiding premature imposition of interpretations on another’s experience, facilitating the acquisition of information and self-awareness, and offering practical assistance played pivotal roles in their perceived support. The value of connecting with individuals who had navigated similar experiences, such as through integration groups, was also emphasised by participants.

The most widely used individual coping strategy was meditation/prayer, followed by cognitive strategies like cultivating acceptance, positive or compassionate self-talk and cognitive distancing. This aligns with models of psychedelic integration that emphasise cognitive behavioural approaches like Acceptance and Commitment Therapy, as proposed by Sloshower et al. (2020) and Watts and Luoma (2020). It also fits with Brook’s (2021) finding that, among those who report undergoing ‘spiritual emergencies,’ the most helpful practices were cultivating compassion, acceptance, gratitude and humility. The similarities between these coping methods and the ongoing efforts to include compassion-and meditation-based strategies in psychedelic preparation protocols (e.g., McAlpine et al., 2024), as a means to improve the integration process, emphasise the importance of integrating contemplative techniques when addressing extended difficulties following psychedelic experiences. The reported efficacy of distraction techniques, such as engaging in gaming, listening to music, or immersing in work align with findings from other contexts. For instance, Iyadurai et al. (2018) highlight the utility of gaming as a distraction technique to manage intrusive thoughts following traumatic events. This observation suggests a broader applicability of such strategies in psychological coping mechanisms.

Several coping techniques reported by survey participants were grounded in embodied, somatic, or physical practices, including breathing exercises, yoga, tai chi, physical exercise, bathing, and rest. These findings align with Gorman et al.’s (2021) integration model, as well as the embodied dimension emphasised in Watts and Luoma’s (2020) ACE model of psychedelic therapy and integration. Additionally, respondents identified spending time in natural environments as beneficial and healing, a concept also underscored by Gandy et al. (2020), Watts and Luoma (2020), and Brook (2021). Consistent with the research of Evans and Read (2020), it is noteworthy that embodied practices appear to facilitate the integration of ‘spiritual emergencies,’ whether induced by psychedelics or other factors.

Respondents also mentioned the value of journaling and self-education in coping with their post-psychedelic difficulties. This fits with Gashi et al.’s (2021) emphasis on narrative practices as central to the integration of challenging psychedelic experiences. In Brook’s (2021), Evans and Read’s (2020), and Corneille and Luke’s (2021) studies of people reporting ‘spiritual emergencies,’ participants also suggested reading and meaning-making were crucial practices for making sense of a powerful yet confusing experience and integrating it into their life. While spiritual interpretations of psychedelic experiences are common, not all psychedelic-related problems are interpreted as spiritually salient, and some participants actively rejected a spiritual interpretation of their difficulties.

Some respondents suggested that their post-psychedelic difficulties were best resolved by a subsequent psychedelic experience. This aligns with Evans & Read’s findings that people sometimes recover from ‘spiritual emergencies’ by re-entering altered states, re-experiencing the trauma of their initial challenging experience, but this time emerging with the feeling they have managed the experience better and not been overwhelmed by it (Evans and Read, 2020). Other respondents reported that they used alcohol and recreational drugs, even in some cases in amounts described as excessive. This reflects the key point that respondents were describing what they found to be helpful at the time, which may or may not be helpful from an expert or therapeutic perspective, or be helpful in the medium or long term.

The data also provides a sense of the challenges of coping with post-psychedelic difficulties. Respondents mentioned a lack of information on post-psychedelic difficulties and a lack of qualified support to deal with them. This accords with Cowley-Court et al.’s (2023) paper on post-ayahuasca integration, where some interviewees spoke of feeling unsupported by the retreat centres they attended, and disconnected from the social environments to which they returned.

The contemporary Western cultural context lacks a comprehensive consensus on the understanding of adverse psychedelic experiences or post-psychedelic difficulties (Breeksema et al., 2022), how to interpret and diagnose them, or even an agreed terminology for them. This lack of consensus may contribute to the wide variety of coping strategies employed to help resolve the difficulties, as individuals pursue possible solutions and resolutions with the varied resources or viewpoint at their disposal.

Given the complex and multifaceted nature of the coping strategies reported in this survey, a model of optimal coping that could be incorporated into integration models or psychedelic-assisted therapy will be one that is sensitive to individual, social and cultural contexts. Individuals exhibit varied responses to different frames and interventions. Diverse factors such as personality types, cultural backgrounds, the nature of challenges encountered, and the stage of recovery may influence the efficacy of various techniques. Therefore, therapists, psychiatrists, peer groups, and integration coaches must adopt a tailored approach, taking into account the individual’s unique characteristics, coping resources, and the cultural framework that resonates most with their current experience. Notably, researchers have emphasised the importance of maintaining metaphysical neutrality when assisting individuals during or after psychedelic experiences (Letheby, 2021). Achieving this balance is a delicate endeavour, particularly considering that a significant portion of extended difficulties encompasses existential and ontological concerns (Evans et al., 2023).

### Limitations and further research

The study has a number of limitations. The sample was mainly White, university educated, and from Western countries. Participants were required to speak English to participate, which means that while the sample included over 40 countries where English is not the native language, potential participants were limited to those in the country who could speak English, which would likely be the more educated individuals in the society. The nature of the sample means that the findings should be interpreted through the lens of psychedelic usage in cultures, where, in the main, such substances are illegal to possess or supply. Given the importance of the cultural environment to the set and setting of taking a psychedelic, the experience of difficulties and the attempt to cope with them should be seen through the lens of a broader cultural environment where a certain amount of concealment/secrecy, and potential for shame, is inherent in the usage of psychedelics (Dupuis, 2022).

The study’s survey methodology allowed for a broad geographical reach of participants. The purposive selection of individuals who reported extended difficulties (who in typical random samples represent the minority) means that this study is aiming to understand this specific group, and makes no claims about how often difficulties or coping strategies occur in the general psychedelic-taking population in any particular culture. What it does is elucidate the kinds of ways that people with such difficulties aim to cope and integrate and which strategies are most commonly found to be helpful. It is possible that participants are erroneously linking their difficulties with the psychedelic experience, and this is not an issue that the current study can speak to. Only a prospective longitudinal randomised control study could ascertain this with a level of credibility, ideally with a control group that undergoes a non-pharmacological disruptive and transformative experience. There was no question for participants to specify the types of difficulties for which they found particular coping strategies and support mechanisms beneficial, thus the link between difficulty types and coping strategies remains unexplored. Additionally, the *extent* to which each coping strategy was found helpful was not assessed or quantified, and participants were able to identify multiple techniques without ranking their effectiveness. This limitation makes it challenging to discern which strategies were most efficacious. Future investigations should aim to elicit more granular insights by inquiring about the most helpful coping technique for specific difficulties and the degree to which these techniques mitigated the challenges faced. Such detailed information could lead to more customised support and resource recommendations for individuals following psychedelic use.

Analysis of the brief text data identified 29 distinct coping techniques. While we aimed at themes that were as conceptually distinct as possible, some overlap between the techniques and themes was evident, for example between meditation and embodied contemplative practices, or between physical self-care and body relaxation. These overlaps are inevitable given that coping strategies exist within complex continua of behaviour and experiences rather than in discrete categories. A deeper analysis to discern the behaviours and cognitions contributing to the coping strategies is an important next step, and we aim to do this through in-depth interviews about post-psychedelic difficulties and coping strategies, that gains more in-depth data per participant than was possible with the brief text approach to data collection used here. An important element of this follow-up research, which at the time of writing is ongoing, is that a dialogue is facilitated with participants about their experience. As part of this, where ‘talking therapy’ is cited as beneficial, further investigation into the specific type of therapy, its duration, and the aspects of the therapy that users find most helpful will be sought. Finally, it remains to be seen to what extent some post-psychedelic difficulties like anxiety, derealization or PTSD might be best treated by the traditional therapeutic and psychiatric treatments for these conditions.

## Conclusion

In conclusion, this study provides an overview of the coping strategies individuals have found beneficial for alleviating post-psychedelic difficulties, from the perspective of an English-speaking international sample that is predominantly White, Western and university educated. Reports from participants indicate that some spent months or years seeking information or advice on their post-psychedelic difficulties. This highlights a need for increased research and accessible information in this domain, aiming to alleviate the varied difficulties that individuals reported and tried to cope with. Given the lack of professional support services aimed at helping individuals with post-psychedelic difficulties, individuals in our mainly Western sample may have been more inclined to seek non-professional support sources, including friends, family, meditation, and exercise, and cope individually using *ad hoc* strategies that they found to work. It is our aspiration that this ongoing research will inform and enhance the response strategies of therapists, psychiatrists, psychedelic welfare services, coaches, guides, clinics, and retreat centres, thereby equipping them to offer adept support for individuals who are experiencing extended difficulties in their recovery and integration processes.

## Data availability statement

The datasets presented in this study can be found in online repositories. The names of the repository/repositories and accession number(s) can be found at: Open Science Framework, https://osf.io/9wk47/.

## Ethics statement

The studies involving humans were approved by University of Greenwich Research Ethics Board. The studies were conducted in accordance with the local legislation and institutional requirements. The participants provided their written informed consent to participate in this study.

## Author contributions

OR: Conceptualization, Data curation, Formal analysis, Funding acquisition, Investigation, Methodology, Project administration, Resources, Software, Supervision, Validation, Visualization, Writing – original draft, Writing – review & editing. JE: Conceptualization, Data curation, Formal analysis, Funding acquisition, Investigation, Methodology, Project administration, Resources, Software, Supervision, Validation, Visualization, Writing – original draft, Writing – review & editing. DL: Conceptualization, Formal analysis, Methodology, Writing – original draft, Writing – review & editing. RM: Writing – original draft, Writing – review & editing, Conceptualization, Formal analysis, Methodology, Visualization. AS: Data curation, Formal analysis, Methodology, Writing – original draft, Writing – review & editing. AF: Formal analysis, Methodology, Writing – review & editing. SS: Methodology, Writing – original draft, Writing – review & editing, Formal analysis. EK: Writing – original draft, Writing – review & editing, Project administration. AM-B: Formal analysis, Methodology, Writing – original draft, Writing – review & editing. KM: Conceptualization, Methodology, Writing – original draft, Writing – review & editing. EP: Conceptualization, Writing – original draft, Writing – review & editing.
