# Supplementary material for: Coming back together: a qualitative survey study of coping and support strategies used by people to cope with extended difficulties after the use of psychedelic drugs
Source: Front Psychol. 2024 May 28;15:1369715. doi: 10.3389/fpsyg.2024.1369715 (PMC11166081; doi:10.3389/fpsyg.2024.1369715)
Supplement: Supplementary file 1 [file Data_Sheet_1.DOCX]

How do you describe your gender?

- Male
- Female
- Other
- Prefer not to answer

What is your age?

- 18-24
- 25-34
- 35-44
- 45-55
- Over 55
- Prefer not to answer

What is your ethnic background?

- Asian
- Black
- White
- Hispanic
- Mixed race
- Other
- Prefer not to answer

What’s your nationality?

________________________________________________________________

| 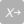 |
| --- |

What’s your highest level of education?

- High school / secondary school
- Bachelor’s degree
- Master’s
- PhD or above
- Other
- Prefer not to answer

| Page Break |  |
| --- | --- |

Please reflect on **one specific psychedelic experience** that led to difficulties or challenges lasting beyond the trip itself.

If you have had multiple psychedelic experiences that have led to post-trip difficulties, please refer to the one that stands out as most difficult for you to integrate/navigate afterwards. 

The following questions focus on the psychedelic experience *itself.* 

| 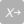 |
| --- |

When did this psychedelic experience happen?

- Within the past 3 weeks
- Within the past month
- Within the past 3 months
- Within the past 6 months
- Within the past year
- 1-3 years ago
- More than 3 years ago

| 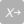 |
| --- |

What substance(s) did you take?

- Ayahuasca
- DMT
- LSD
- Mescaline / Peyote / San Pedro
- Psilocybin / Magic Mushrooms
- Ketamine
- MDMA
- Salvia
- Cannabis
- Other - please specify __________________________________________________

| 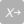 |
| --- |

Did you or someone else present know the dose you were taking?

- Yes
- No

| 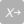 |
| --- |

Where did this psychedelic experience happen?

- At a clinic or medical trial
- At an underground psychedelic therapy session
- On a psychedelic retreat
- In a group ceremony
- At a rave, nightclub or festival
- At a party
- With a friend, partner, or group of friends
- On my own
- Other __________________________________________________

How challenging was the psychedelic experience itself?

|  | Not at all Challenging | Moderately Challenging | Very Challenging | Extremely Challenging |
| --- | --- | --- | --- | --- |

| “The psychedelic experience was..." | 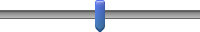 |
| --- | --- |

| Page Break |  |
| --- | --- |

The following questions explore the **difficulties or challenges** you experienced *after* your psychedelic trip.

| 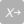 |
| --- |

Which of the following options best describe the difficulties/challenges you experienced after your psychedelic trip. Please select all that apply to you.

- Social difficulties (e.g., the way you interacted the related to other people)
- Perceptual difficulties (e.g., the way your vision functioned)
- Cognitive difficulties (e.g., the way you thought about things)
- Emotional difficulties (e.g., the way you felt emotionally or the ability you had to emotionally regulate)
- Spiritual difficulties (e.g., your spiritual beliefs)
- Ontological difficulties (e.g., the way you understood reality and existence)
- Self-perception difficulties (e.g., the way you felt about (or understood) your self)
- Other difficulties (please describe briefly) __________________________________________________

How long did the difficulties/challenges last after the trip?

- Less than a week
- Up to a week
- Up to a month
- Up to 3 months
- Up to 6 months
- Up to a year
- 1-3 years
- More than 3 years

| 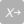 |
| --- |

To what extent do you agree with the following statement: "“The difficulties I experienced during the psychedelic trip contributed to the difficulties I experienced after”

- < Don't Know >
- Strongly Disagree
- Disagree
- Agree
- Strongly Agree

Please describe the lasting difficulties that you encountered after your psychedelic experience. We would like you to write for about 3 to 5 minutes.

________________________________________________________________

________________________________________________________________

________________________________________________________________

________________________________________________________________

________________________________________________________________

| Page Break |  |
| --- | --- |

The following questions focus on **coping strategies and support** that you used in the days/weeks/months **after** the psychedelic experience.

***Data from the following two questions used for the article: “Coming back together: A qualitative survey study of coping and support strategies used by people to cope with extended difficulties after the use of psychedelic drugs”***

If you used coping strategies that you found helpful in dealing with the difficulties/challenges after the trip, please describe these in a short paragraph.

________________________________________________________________

________________________________________________________________

________________________________________________________________

________________________________________________________________

________________________________________________________________

| 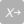 |
| --- |

Did you seek support from other people to help with the difficulties you experienced? If you found some or all of the support from others to be helpful, please describe what you found to be most helpful and why.
________________________________________________________________

________________________________________________________________

________________________________________________________________

________________________________________________________________

________________________________________________________________

To what extent do you agree with the following statement: "The difficulties that this experience caused still affect me negatively."

- < Don't Know >
- Strongly Disagree
- Disagree
- Agree
- Strongly Agree

Do you still take psychedelic drugs?

- Yes
- No

Had you been diagnosed with a mental illness prior to the psychedelic experience?

- Yes
- No

| 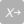 |
| --- |

Do you think this prior diagnosis may be linked to the difficulties you experienced during or after the psychedelic experience?

- Yes
- No
- Not Sure

Was there a traumatic experience in your childhood or youth which you think may have played a role in the difficulties that arose during or after the psychedelic experience?

- Yes
- No
- Not sure

Have you been diagnosed with a mental illness *since*the psychedelic experience?

- Yes
- No

| 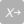 |
| --- |

Do you personally think that your psychedelic experience contributed towards this diagnosis?

- Yes
- No
- Not Sure

Please rate your agreement with the below statement: “I believe that the insights and healings gained from psychedelics, when taken in a supportive setting, are worth the risks involved.”

- Strongly Disagree
- Disagree
- Agree
- Strongly Agree
